# Pre-harvest CaCl₂ foliar application enhances peach quality during cold storage: Insights from response surface modeling

**DOI:** 10.1016/j.fochx.2026.104425

**Published:** 2026-09-26

**Authors:** Isa Hazbawi

**Affiliations:** Department of Biosystems Engineering, Faculty of Agriculture, Lorestan University, Khorramabad, Iran

**Keywords:** CaCl₂, Peach, Post-harvest quality, Cold storage, Nutritional value, Response surface methodology

## Abstract

Peaches (*Prunus persica* L.) are highly perishable, and cold storage often causes quality loss, color changes, and ascorbic acid degradation. This study evaluated the effects of pre-harvest foliar CaCl₂ application (0–3%) on the quality of ‘Zafarani’ peaches during cold storage for 40 days at 1 ± 1 °C and 90 ± 5% RH. CaCl₂ reduced ethylene production and helped maintain fruit quality during cold storage. Response surface methodology (RSM) showed significant effects of CaCl₂ concentration and storage duration on fruit color, acidity, ascorbic acid content, and physiological stability. Pareto analysis indicated calcium as the main factor affecting skin brightness (L^⁎^). The RSM-derived optimal storage strategy (1.96% CaCl₂ for 34.8 days) predicted 0.87% acidity and 27.31 μmol kg^−1^ h^−1^ ethylene production, whereas 2.21% CaCl₂ maximized ascorbic acid (4.20 μg g^−1^). The validation error was <2.78%. Pre-harvest CaCl₂ application effectively preserved selected fruit quality attributes during cold storage.

## 1 Introduction

The Peach (*Prunus persica* L.) is recognized as a stone fruit of high economic importance worldwide. Due to its favorable taste, richness in phenolic compounds, and natural antioxidants, it plays a significant role in both fresh fruit markets and processing industries, while its bioactive compounds also contribute to its potential value as a functional food source (Bento et al., 2022; Chen et al., 2022; Kumari et al., 2023). Despite its high nutritional and economic value, the peach is highly perishable after harvest owing to its soft tissue structure and rapid metabolic activity, which make preserving quality throughout the supply chain a major challenge (Qin et al., 2025; Veerappan et al., 2021).

Peach fruit ripening is a climacteric process characterized by a sudden increase in respiration and ethylene production, which results in tissue softening, flavor deterioration, and reduced marketability (Qin et al., 2025). Biochemical and physiological alterations during ripening affect tissue structure, color, aroma, and the balance of sugars and organic acids, while environmental factors such as temperature and humidity strongly influence the intensity of these processes (Schudel et al., 2023; Veerappan et al., 2021). Therefore, the limited shelf life of fresh peaches stems from their inherent perishability and sensitivity to storage conditions, underscoring the importance of developing effective post-harvest strategies to preserve fruit quality.

Conventional low-temperature storage is the most common approach for slowing metabolic activity and extending the post-harvest life of peaches (Brizzolara et al., 2020; Hayat et al., 2023). However, prolonged storage, even under cold conditions, can induce chilling injury (CI), discoloration, flavor loss, and nutritional degradation (Song et al., 2022; Zhao et al., 2024). Previous studies have indicated that effective temperature management during storage and the regulation of pre-harvest conditions—such as the application of calcium chloride (CaCl₂)—can enhance the efficacy of cold storage while reducing CI and premature ripening (Guo et al., 2023; Song et al., 2022). The commercially important ‘Zafarani’ peach cultivar also exhibits limited storability, primarily because of rapid softening, firmness loss, weight loss, and increased decay during cold storage, which collectively reduce its marketability and storage potential (Rahimi et al., 2019; Askari Sarcheshmeh et al., 2020).

Several preharvest treatments, including salicylic acid (SA), methyl jasmonate, and CaCl₂, have been investigated to improve peach fruit quality. Among these, pre-harvest CaCl₂ application has received particular attention due to its critical role in maintaining cell wall integrity, delaying fruit softening, and mitigating chilling injury (Ali et al., 2021; Huan et al., 2022; Souza et al., 2023). Previous studies have used different preharvest CaCl₂ spray schedules, including applications initiated at different phenological stages or repeated during fruit development, resulting in variable responses among cultivars. Consequently, an optimal preharvest application schedule has not yet been conclusively established (Carrasco-Cuello et al., 2024; Shahid et al., 2020).

Calcium is a key element contributing to cell wall strength and plasma membrane stability; its role in reducing tissue softening is attributed to the inhibition of cell wall–degrading enzymes such as polygalacturonase and pectinesterase (Brummell et al., 2022; Xu et al., 2022). Numerous studies have demonstrated that pre- and post-harvest treatments with CaCl₂ not only maintain tissue firmness but also reduce decay incidence, lower the respiration rate, and preserve phenolic compounds, vitamins, and other nutritional indicators in various fruit species (Erogul et al., 2024; Guo et al., 2023; Lv et al., 2020). Specifically, pre-harvest foliar CaCl₂ application delays climacteric ripening by reducing ethylene production and softening rates, thereby maintaining fruit quality during cold storage.

Despite considerable progress, inconsistent responses to preharvest CaCl₂ application occur due to variations in cultivars, environmental conditions, and spray schedules (Carrasco-Cuello et al., 2024). High concentrations of CaCl₂ may cause surface injury and skin discoloration, whereas low concentrations are often ineffective (Maletsika et al., 2023; Shin et al., 2023). Moreover, the response of peaches to calcium varies depending on the cultivar, environmental conditions, and fruit maturity stage (Carrasco-Cuello et al., 2024). Therefore, further studies are still needed to optimize the application strategy for different cultivars and production conditions.

Modern statistical approaches, such as response surface methodology (RSM), are effective tools for determining the optimal conditions of calcium application and fruit storage duration (Castro et al., 2021; Javed et al., 2025). RSM enables the simultaneous evaluation of multiple factors and their interactions and facilitates the identification of optimal factor combinations. Compared with conventional parametric approaches, RSM provides a systematic framework for modeling response surfaces and optimizing multiple responses, making it suitable for studies involving interacting pre- and post-harvest factors (Hazbawi et al., 2026; Kovalchuk et al., 2017; Kovalchuk et al., 2018). However, most previous studies have primarily focused on single-factor approaches or treated pre-harvest applications and post-harvest storage as isolated events, leaving a gap in understanding their interactive dynamics. The novelty of this study lies in applying RSM to simultaneously optimize pre-harvest CaCl₂ concentration and post-harvest storage duration to predict multiple quality attributes of ‘Zafarani’ peach fruits during cold storage.

Accordingly, the present study was designed to investigate and optimize the effects of pre-harvest foliar CaCl₂ application and storage duration on the qualitative, physiological, and nutritional attributes of this cultivar. Specifically, this research aimed to determine the optimal CaCl₂ concentration and assess its effectiveness across different storage periods in preserving fruit quality, color, and nutritional attributes under cold storage conditions.

## 2 Materials and methods

### 2.1 Fruit harvesting and post-harvest storage conditions

The experimental design integrated both field (pre-harvest) and laboratory (post-harvest) phases. The field experiment was conducted in a commercial peach orchard located in Khorramabad, Iran. To prepare the treatment solutions, CaCl₂ powder (Merck, Germany) was dissolved in distilled water, and Tween 20 surfactant (0.1% *v*/v) was added as a non-ionic surfactant to reduce surface tension and enhance solution spreading and canopy absorption without inducing phytotoxicity. Forty 10-year-old ‘Zaferani’ peach trees characterized by uniform canopy structures and a 7-m planting spacing in each direction were initially monitored. From this population, 29 trees with the most uniform vegetative characteristics were selected for the experimental runs. Each tree served as an independent biological replicate and represented the experimental unit. This cultivar was chosen for its high economic value, desirable yield, and significant contribution to export markets, making it one of the major commercial peach cultivars in Iran.

Foliar applications were executed at three critical fruit development stages: cell division (30 days after full bloom; DAFB), pit hardening (60 DAFB), and cell expansion (90 DAFB). The treatments consisted of three CaCl₂ concentrations: 0, 1.5, and 3% (*w*/*v*). Solutions were applied onto the leaves and fruit surfaces until runoff using a motorized backpack sprayer with an application volume of approximately 2 L per tree. Tween-20 (polyoxyethylene sorbitan monolaurate) was used as a surfactant to reduce the surface tension of the solution, which has been documented as a safe concentration without inducing phytotoxicity on peach. The field experiment was conducted according to the 13 experimental runs generated by the face-centered central composite design (FCCCD), comprising eight non-center runs with three independent trees per run and one center condition replicated independently on five trees. Thus, the eight non-center runs involved 24 independent trees, while the five center-point replicates involved five additional independent trees, resulting in 29 experimental trees in total. At each storage interval (0, 20, and 40 days), five fruits were randomly collected from the mid-height peripheral canopy of each experimental tree. Accordingly, each tree contributed five fruit subsamples, resulting in 145 sampled fruits across the complete experimental design. The five fruits collected from each tree were treated as subsamples rather than independent biological replicates. The selected range of CaCl₂ concentrations was based on previous studies identifying this interval as an effective and safe range for maintaining fruit quality without inducing phytotoxic effects (Guo et al., 2023; Souza et al., 2023; Mu et al., 2026; Hou et al., 2021). Accordingly, three concentration levels within this range were selected, as they provided sufficient factor levels for fitting the second-order response surface methodology (RSM) model and reliably evaluating the studied responses within the experimental design (Akbari et al., 2026; Hazbawi, 2026; Hazbawi & Etezadi, 2026).

At the physiological maturity stage, which was determined based on the change in the fruit background color from green to yellow, a flesh firmness of 70–80 N, and a total soluble solids (TSS) content of 10–11°Brix, uniform, healthy, and undamaged fruits from each treatment were harvested and transferred to the laboratory. These maturity indices are consistent with the established commercial harvest criteria for the ‘Zafarani’ peach cultivar and with the commercial harvest stage identified by the experienced local orchardists. The samples were subsequently stored in a cold room at 1 ± 1 °C and 90 ± 5% RH for 40 days. Qualitative, physiological, and nutritional attributes were evaluated at three intervals: 0, 20, and 40 days of storage. Independent fruit samples were assessed at each storage interval; therefore, the same fruits were not repeatedly measured across storage times. For each experimental run × storage combination, the fruits collected from the corresponding experimental trees constituted the predefined sample pool for that storage interval. From this pool, three fruits were used for each destructive analytical determination, including titratable acidity (TA), ethylene production (ET), and ascorbic acid (AsA), while non-destructive measurements, such as surface color, were performed on the corresponding fruit samples. The monitored parameters included titratable acidity (TA), surface color, ethylene production (ET), and ascorbic acid (AsA) content (Fig. 1).

**Fig. 1:**
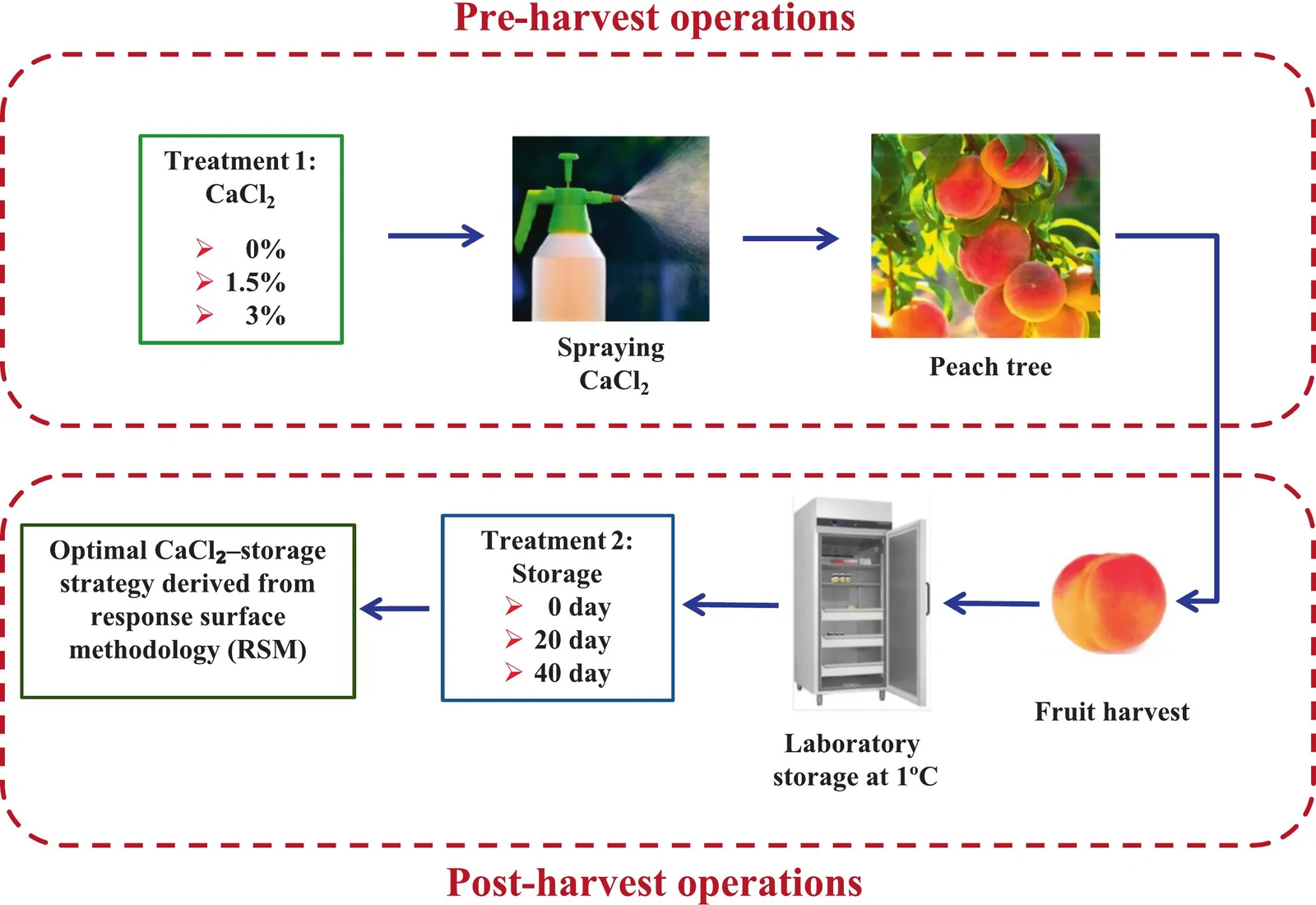
Diagram illustrating the effect of CaCl₂ treatment on the nutritional properties of peach fruit during storage.

### 2.2 Determination of fruit surface color

The surface color characteristics of peach fruits were determined using the CIE color system parameters, including L^⁎^ (lightness), a^⁎^ (negative values indicate green and positive values indicate red), and b^⁎^ (negative values indicate blue and positive values indicate yellow), using a portable chromameter (model CR-400, Konica Minolta, Japan) (Mrazova et al., 2021).

### 2.3 Determination of titratable acidity (TA)

For TA determination, 10 mL of peach juice obtained from a composite sample of three fruits was diluted with 40 mL of distilled water. Subsequently, 10 mL of the diluted solution was titrated with 0.1 N NaOH until the endpoint pH of 8.2 was reached, using phenolphthalein as the color indicator. TA was expressed as malic acid equivalents (%) and calculated using the equation TA (%) = (V × 0.0067 / Vₛ) × 100, where V is the volume (mL) of 0.1 N NaOH consumed and Vₛ is the volume (mL) of the sample used for titration. The analysis was performed using three independent composite samples (Kotani et al., 2020).

### 2.4 Determination of ascorbic acid (AsA)

To quantify the concentration of ascorbic acid, 2.5 g of the edible portion of the fruit was thoroughly homogenized with 10 mL of a 16% metaphosphoric acid solution containing 0.18% disodium EDTA. The resulting suspension was centrifuged at 5000 ×*g* for 10 min and subsequently incubated at 23 ± 1 °C for 10 min. The absorbance of the clear supernatant was measured at 760 nm using the Folin–Ciocalteu colorimetric method (Abera et al., 2020). l-Ascorbic acid was used to prepare the calibration curve over the concentration range of 5–125 μg mL^−1^, with a coefficient of determination (R^2^) of 0.983. The method showed approximately 100% recovery, with no significant interference from the tested compounds. Analytical precision was evaluated in terms of repeatability and intermediate precision, yielding RSD values of 0.5–2.4% and 1.1–3.8%, respectively. The final ascorbic acid content was calculated from the calibration curve and expressed as micrograms per gram of fresh weight.

### 2.5 Measurement of ethylene production (ET)

To evaluate the ET rate, three peach fruits were randomly selected from each replicate and weighed. The samples were placed in an airtight container with a rubber-sealed lid for one hour to allow ethylene accumulation in the headspace. The container had a total volume of 3.2 L. At the end of the incubation period, 1 mL of the headspace gas was withdrawn using a gas-tight syringe and directly injected into a gas chromatograph (model 6890 N: Agilent Technologies, Palo Alto, CA, USA) equipped with a flame ionization detector (FID) and an HP-PLOT Q capillary column (30 m × 0.53 mm × 40 μm). Nitrogen (N₂) was used as the carrier gas at a flow rate of 30 mL min^−1^. For quantitative analysis, the system was calibrated using a six-point external calibration curve with ethylene standards at 0.1, 0.5, 1.0, 2.0, 3.0, and 5.0 μL L^−1^, yielding good linearity (R^2^ = 0.988). The limit of detection (LOD) and limit of quantification (LOQ) were 0.05 and 0.15 μL L^−1^, respectively, calculated from the standard deviation of the response and the slope of the calibration curve. Analytical precision was assessed using repeated measurements, with intra-day and inter-day RSD values of 1.3–2.2% and 2.1–3.4%, respectively. The retention time of ethylene was approximately 2.5 min under the described chromatographic conditions. The temperatures of the column, injector, and detector were set at 110, 100, and 250 °C, respectively, to ensure optimal separation and detection conditions. The ET rate was expressed as μmol kg^−1^ h^−1^ (Alvarez-Hernandez et al., 2020).

### 2.6 Experimental design and data analysis

The present study was conducted to investigate the effects of optimal CaCl₂ solution concentrations and storage duration on the nutritional and physical properties of *Prunus persica* (cv. Zaferani). An RSM framework was employed for experimental design and data analysis. The independent variables included CaCl₂ concentration (0, 1.5, and 3%) and cold storage duration (0, 20, and 40 days), whose effects on fruit L^⁎^, TA, a^⁎^/b^⁎^, AsA, and ET were evaluated. Data were analyzed using a second-order regression model (Eq. 1) to precisely determine the relationships between the independent variables and the corresponding responses (Hazbawi & Safaeinezhad, 2023).

(1) $$Y=B_{0}+B_{1}X_{1}+B_{2}X_{2}+B_{3}X_{1}X_{2}+B_{4}X_{1}^{2}+B_{5}X_{2}^{2}$$

The response variable (*Y*) was modeled as a function of the CaCl₂ concentration (*X₁*) and storage time (*X₂*). The estimated coefficients—including the intercept (*B₀*), main effects (*B₁*, *B₂*), interaction effect (*B₃*), and quadratic effects (*B₄*, *B₅*)—were used to analyze the magnitude and direction of the factors' influence on the responses.

To simulate and optimize the fruit treatment process, RSM combined with a face-centered central composite design (FCCCD; α = 1) was applied using Design-Expert software (version 11). The FCCCD consisted of four factorial points, four axial points, and five center points, resulting in a total of 13 experimental runs. Because α = 1, the axial points coincided with the factorial boundaries. The complete design matrix, including the actual and coded factor levels, is presented in Table 1, and the experimental run order was randomized by the Design-Expert software. The eight non-center design runs were each conducted using three independent biological replicates (trees), whereas the center condition was independently replicated using five trees. Thus, 24 independent trees were used for the eight non-center runs and five independent trees for the center condition, resulting in 29 experimental trees in total. For each run, five fruits were sampled from each tree as subsamples, and the tree—not the individual fruit—was considered the experimental unit.

**Table 1:** Experimental design matrix of the face-centered central composite design (FCCCD), including coded and actual factor levels.

Run	Type	CaCl₂ concentration (%)	Storage duration (day)	Coded C	Coded S
1	Center	1.5	20	0	0
2	Center	1.5	20	0	0
3	Center	1.5	20	0	0
4	Center	1.5	20	0	0
5	Center	1.5	20	0	0
6	Axial	1.5	40	0	1
7	Axial	3	20	+1	0
8	Axial	1.5	0	0	−1
9	Axial	0	20	−1	0
10	Factorial	3	0	+1	−1
11	Factorial	0	0	−1	−1
12	Factorial	3	40	+1	1
13	Factorial	0	40	−1	1

**Note:** The FCCCD was face-centered (α = 1); therefore, the axial points coincided with the factorial boundaries. C represents CaCl₂ concentration and S represents storage duration. The experimental run order was randomized using Design-Expert software (version 11).

Accordingly, response values for each non-center run were calculated from three independent tree-level replicates, whereas the five center-point runs represented five independent biological replicates of the center condition. The five independent center-point replicates were included to provide an estimate of pure experimental error, rather than representing repeated measurements of the same biological material. The effects of the independent variables on the response parameters were statistically analyzed at a 95% confidence level (*p* < 0.05). All prespecified linear, interaction, and quadratic terms of the second-order RSM models were retained because they were statistically significant for the evaluated responses; therefore, no model terms were removed. The statistical significance of the fitted models and their individual terms is provided in Supplementary Table S1. Based on the data presented in Table 2, along with the defined process constraints and objectives, optimal CaCl_2_ treatment conditions were determined, aiming to identify conditions associated with preservation of nutritional and visual quality during cold storage. Furthermore, Pearson's correlation analysis was conducted using R software (version 2022) to evaluate the linear relationships and the degree of dependency among the response variables.

**Table 2:** Parameter ranges and boundary conditions applied.

Parameter	Unit	Symbol	Min	Max	Target
Calcium chloride (CaCl_2_)	%	C	0	3	In range
Storage	days	S	0	40	Maximum
Color lightness	–	L^⁎^	52.01	69.03	Control value
Titratable acidity	%	TA	0.65	1.25	Control value
Color ratio	–	a^⁎^/b^⁎^	0.25	0.65	Control value
Ethylene production	μmol kg^−1^ h^−1^	ET	9.01	37.03	Control value
Ascorbic acid	μg g^−1^	AsA	2.4	4.2	Control value

## 3 Results and discussion

### 3.1 Statistical analysis of correlation coefficients among independent variables

Pearson's correlation analysis revealed that a^⁎^/b^⁎^ was significantly and negatively correlated with AsA and TA (*r* = −0.85 and *r* = −0.64, respectively), indicating that an increase in skin redness was associated with a decrease in antioxidant content and acidity (Fig. 2). Moreover, a^⁎^/b^⁎^ showed a significant positive correlation with ET (*r* = 0.50, *p* < 0.05), indicating an association between increased redness and ethylene production during fruit ripening. These strong correlations, particularly between a^⁎^/b^⁎^ and AsA and between a^⁎^/b^⁎^ and ET, highlight the close relationship between fruit color development, nutritional status, and ripening-related physiological activity and provide a basis for considering these responses in the subsequent RSM optimization. TA was also found to be negatively correlated with ET, while L^⁎^ exhibited a positive correlation with AsA, suggesting a coordinated relationship between physical attributes and hormonal activity during fruit ripening. Other relationships, such as those between ET and AsA, L and a^⁎^/b^⁎^, or TA and L^⁎^, were not statistically significant, revealing the complexity of interactions among fruit traits. The reported Pearson's correlation *p*-values are unadjusted (raw) *p*-values; no correction for multiple comparisons was applied because the correlation analysis was intended as an exploratory assessment of linear associations among the measured response variables rather than as a confirmatory hypothesis-testing procedure. These findings highlight that color, acidity, and hormonal factors mutually influence one another, and understanding these interactions can play a crucial role in determining the optimal harvest time and managing post-harvest conditions to preserve quality, extend shelf life, and enhance the market value of fresh fruits.

**Fig. 2:**
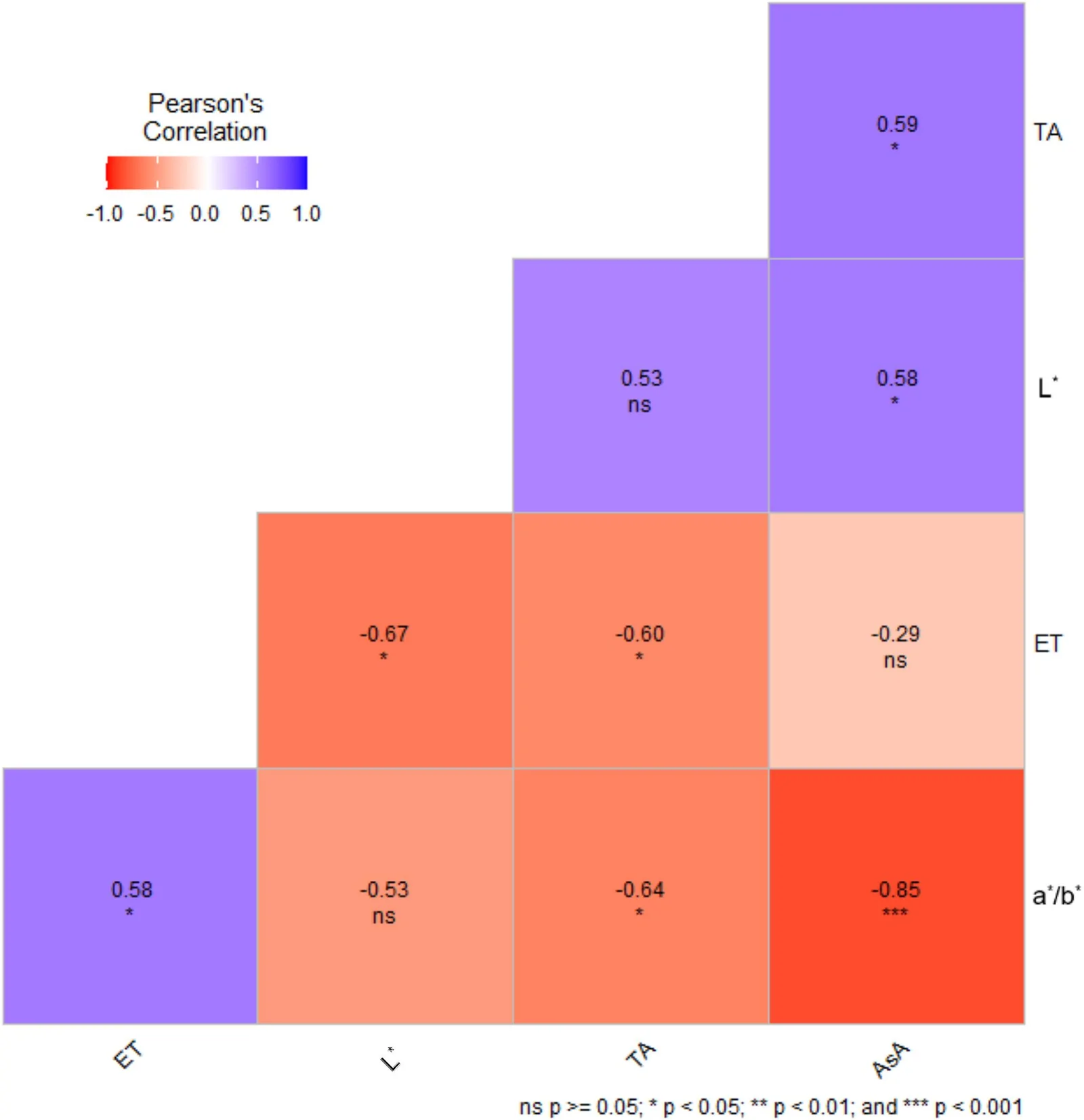
Pearson correlation among color lightness (L^⁎^), titratable acidity (TA), color ratio (a^⁎^/b^⁎^), ascorbic acid (AsA), and ethylene production (ET) in CaCl₂-treated peach fruits during storage.

### 3.2 Key factors in modeling the effect of pre-harvest foliar CaCl₂ spray on the quality preservation of peach fruit

The statistical analysis results, presented in Table 3, indicate that both CaCl₂ concentration and storage duration exert significant effects on the evaluated quality parameters. The fitted response surface models showed high explanatory power, with R^2^ values of 0.927 for L^⁎^, 0.949 for TA, 0.981 for a^⁎^/b^⁎^, 0.986 for ET, and 0.929 for AsA. Adjusted R^2^ and predicted R^2^ values were in reasonable agreement for all responses, with differences of less than 0.2, consistent with the model adequacy criterion reported by Design-Expert. In addition, all adequate precision values were substantially greater than 4, meeting the Design-Expert criterion for an adequate signal-to-noise ratio, while the coefficient of variation (CV) values were within an acceptable range (Table 3). Residual diagnostic plots showed no apparent systematic patterns, supporting the adequacy of the fitted models. The statistical significance of the RSM model terms was assessed using the model-based ANOVA without adjustment for multiple comparisons, as the terms were evaluated within the prespecified second-order response surface model rather than as independent exploratory tests. This performance verifies that the developed regression models are not only reliable for analyzing the relationships between the independent variables and responses but also support their potential applicability to practical post-harvest management decisions, such as optimizing the CaCl₂ concentration to preserve quality and extend the shelf life of peaches under real storage conditions.

**Table 3:** Predicted response behavior based on nonlinear regression analysis.

Parameter	Model	R^2^	Adjusted R^2^	Predicted R^2^	Adequate precision	CV (%)
L^⁎^	62.79 + 4.33C – 1.67S + 4.25C × S – 2.47C^2^ + 2.55S^2^	0.927	0.876	0.740	15.89	2.53
TA	0.922 + 0.138C – 0.140S – 0.073C × S – 0.108C^2^ + 0.067S^2^	0.949	0.913	0.747	17.45	5.20
a^⁎^/b^⁎^	0.326–0.047C + 0.137S – 0.060C × S + 0.041C^2^ + 0.061S^2^	0.981	0.967	0.881	29.10	5.72
ET	28.38–3.33C + 8.84S – 5.01C × S + 2.18C^2^ – 10.31S^2^	0.986	0.977	0.890	30.49	5.53
AsA	4.06 + 0.355C – 0.345S + 0.250C × S – 0.360C^2^–0.430S^2^	0.929	0.878	0.721	13.86	5.01

**Note:** C: CaCl₂ concentration (%); S: Storage duration (days); R^2^: Coefficient of determination; CV: Coefficient of variation.

The Pareto analysis revealed that the pre-harvest CaCl₂ treatment (C) played the most significant role in maintaining the brightness of peach skin and retarding visual senescence by preventing discoloration (Fig. 3a). Across the five responses, the relative contribution of model terms showed a distinct response-dependent pattern: L^⁎^ and TA were primarily influenced by linear effects of C and S, whereas the quadratic storage effect (S^2^) became more prominent for a^⁎^/b^⁎^, ET, and AsA; the quadratic CaCl₂ effect (C^2^) also contributed notably to AsA. This shift from predominantly linear to stronger quadratic effects indicates that the influence of CaCl₂ and storage duration was response-specific and nonlinear for several quality attributes. The interaction between the pre-harvest foliar CaCl₂ treatment and cold storage (C × S) further enhanced brightness, highlighting the importance of synchronizing treatment and storage conditions. In contrast, the duration of cold storage (S) and the quadratic effect of the CaCl₂ concentration (C^2^) resulted in a decline in this parameter and overall visual quality. TA decreased with prolonged storage time (Fig. 3b), which can be attributed to the consumption of organic acids during respiration; however, the quadratic effect of storage duration (S^2^) partially mitigated this decline. The a^⁎^/b^⁎^ ratio was predominantly influenced by the positive effect of cold storage duration, indicating increased redness and ripening (Fig. 3c), while the negative interaction between the CaCl₂ treatment and storage (C × S) somewhat limited pigment formation. ET increased with extended storage time (Fig. 3d), though the quadratic effect of storage (S^2^) moderated this rise, and the CaCl₂ treatment contributed to improved ripening control and maintenance of physiological stability. Ultimately, the CaCl₂ treatment helped preserve higher levels of vitamin C, whereas cold storage was identified as the main factor responsible for its reduction (Fig. 3e). Therefore, precise adjustment of the CaCl₂ concentration and storage duration is considered essential for maintaining the visual quality and nutritional content, as well as for delaying senescence in peaches.

**Fig. 3:**
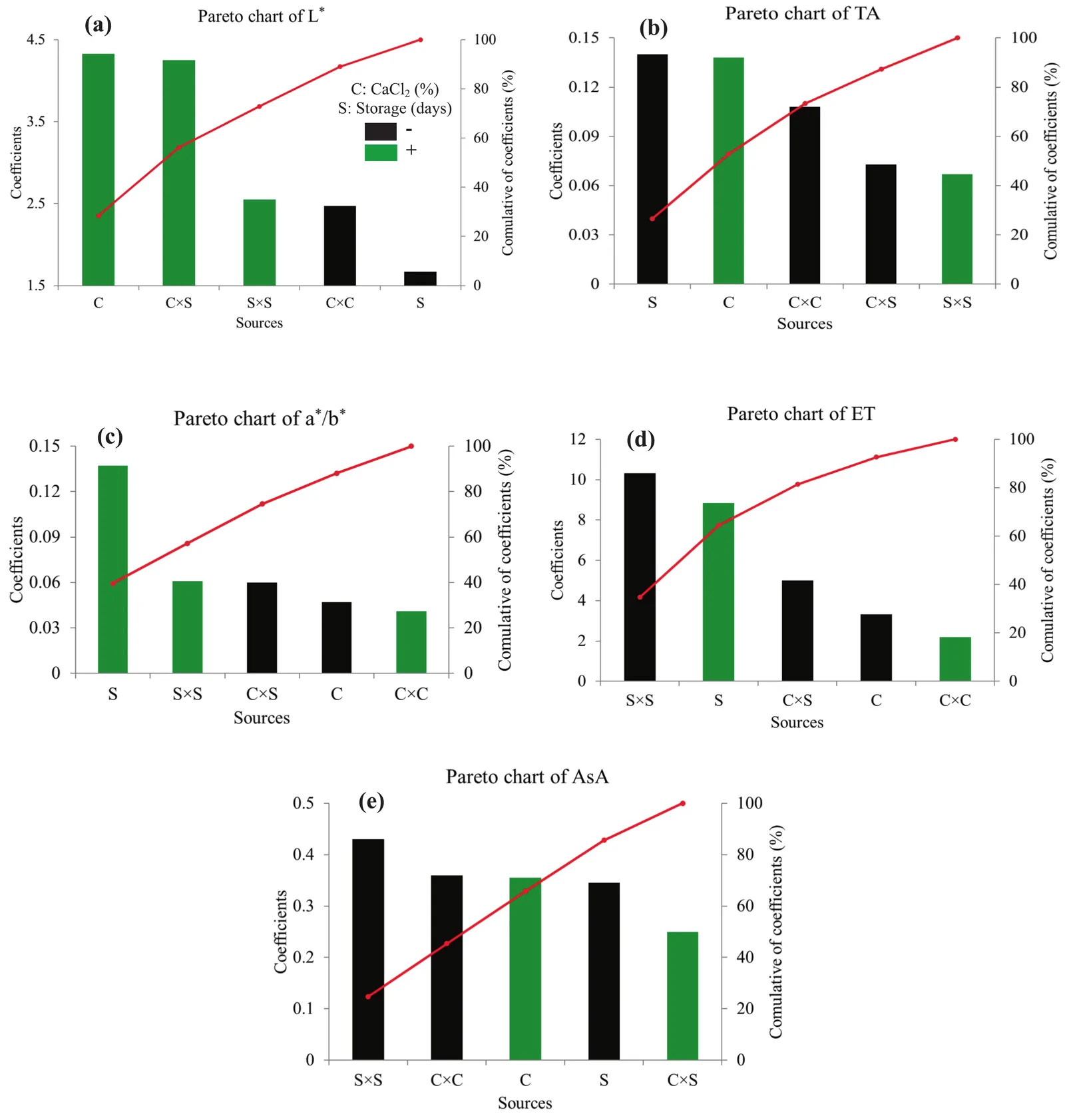
Pareto analysis of regression coefficients for nutritional and appearance quality indicators of peach under CaCl₂ treatment and storage. C: CaCl₂ (%); S: Storage (days); black: negative (−) effect; green: positive (+) effect. (For interpretation of the references to color in this figure legend, the reader is referred to the web version of this article.)

### 3.3 L^⁎^ index

In the evaluation of fruit appearance quality, L^⁎^ is considered one of the key parameters determining freshness and consumer acceptability (Hazbawi, Jahanbakhshi, & Sepehr, 2025). Based on the second-order polynomial regression model, a significant correlation (*p* < 0.05) was observed between peach fruit L^⁎^ and two factors: CaCl₂ concentration and cold storage duration, as well as their interaction (Table 3). Data analysis indicated that the L^⁎^ value of untreated fruits was 52.01 after 40 days of storage, whereas that of fruits treated with 3% CaCl₂ was 69.03 under the same storage duration. Although the difference between treatments was relatively large, higher L^⁎^ values in CaCl₂-treated peaches have also been reported under shorter storage periods and comparable or lower CaCl₂ concentrations (Ilahi et al., 2026). Importantly, the higher L value observed with 3% CaCl₂ after 40 days should not be interpreted as a general brightening effect of prolonged storage; rather, it reflects a CaCl₂-dependent modification of storage-related changes in lightness. This response may be associated with the ability of calcium treatment to reduce storage-related browning and maintain tissue integrity (Han et al., 2023; Xie et al., 2021); however, these mechanisms were not directly evaluated in the present study. The high coefficient of determination (R^2^) confirmed the model's strong predictive capability for changes in peach lightness (Table 3).

The response surface plot revealed that the interaction between CaCl₂ concentration and cold storage duration had a substantial effect on variations in the L^⁎^ index (Fig. 4). The highest L^⁎^ value was obtained when both factors were at their maximum levels (3% CaCl₂ concentration and 40 days of storage), whereas the lowest values were observed under a low CaCl₂ concentration and extended storage time. This finding should be interpreted as a CaCl₂-dependent modification of the storage response rather than as an increase in brightness caused by prolonged storage. In particular, the marked decline in L observed in untreated fruit during storage was substantially attenuated at higher CaCl₂ concentrations. These findings suggest that the optimized application of calcium treatment, combined with proper cold storage management, can contribute to the preservation of the measured visual quality attributes, potentially through mechanisms reported in previous studies (Loay et al., 2021); however, these mechanisms were not directly evaluated in the present study.

**Fig. 4:**
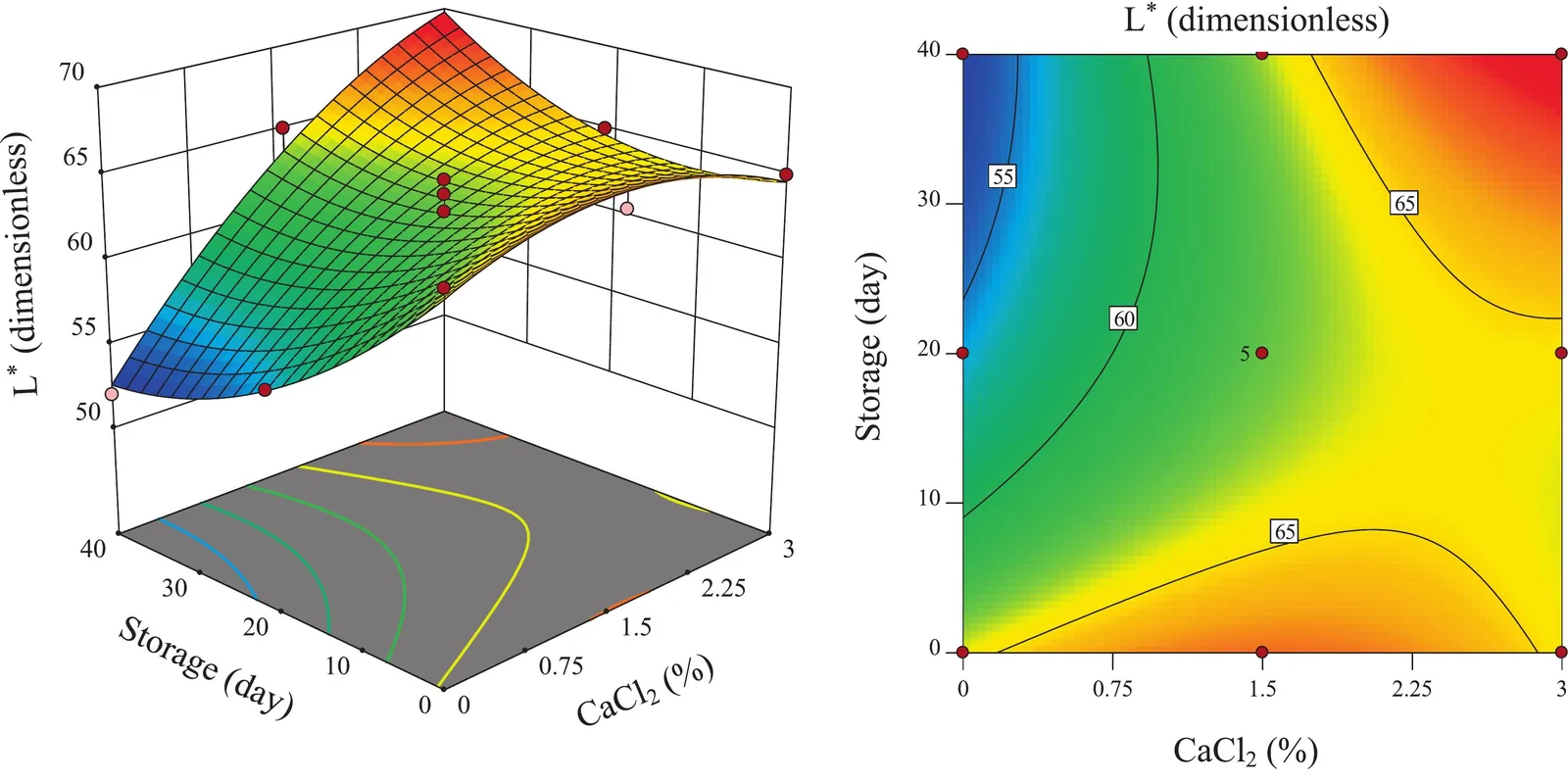
Response surface and contour plots showing the combined influence of CaCl₂ treatment and cold storage on the L^⁎^ value of peaches.

During cold storage, the L^⁎^ index of peach fruit generally decreased under the control and lower CaCl₂ treatments, which is often associated with complex biochemical alterations (Fig. 4). This reduction has been widely attributed in previous studies to chlorophyll degradation through enzymes such as chlorophyllase and peroxidase, along with increased activities of polyphenol oxidase and peroxidase, which oxidize phenolic compounds into quinones and subsequently melanins, thereby intensifying surface browning and decreasing light reflectance (Gohari et al., 2021; Jiang et al., 2023). These processes are generally triggered by chilling stress and cellular senescence, resulting in diminished visual quality, texture modification, loss of volatile compounds, and reduced nutritional value of the fruit (Franzoni et al., 2023; Liu et al., 2022). The decline in lightness and the occurrence of browning during cold storage are generally considered to reflect a complex interaction among physiological stress, enzymatic activity, and oxidative reactions, the control of which is essential for maintaining fruit quality.

The application of pre-harvest foliar CaCl₂ has been found to alleviate the adverse effects of long-term storage (Fig. 4). At the early stages, low concentrations of CaCl₂ exert minimal influence on lightness; however, at later stages, higher concentrations effectively attenuated the storage-related decline in the L^⁎^ index. According to previous studies, this effect may be associated with calcium's ability to stabilize the cell wall structure through pectic cross-linking, which has been reported to inhibit PPO activity, and enhance antioxidant enzyme functions, thereby suppressing browning (Ahmad et al., 2024; Erogul et al., 2024; Souza et al., 2023). Furthermore, previous studies suggest that calcium-mediated pectin–polyphenol interactions and improved cellular integrity may reduce oxidative damage from reactive oxygen species and extend the fruit's shelf life (Guo et al., 2023; Hu et al., 2023). These proposed mechanisms may contribute to the observed preservation of L^⁎^; however, they were not directly evaluated in the present study. Consistent with this interpretation, previous studies in peaches have reported that CaCl₂ treatment helped maintain higher L values during storage, indicating a delay in storage-related discoloration rather than a direct brightening effect (Han et al., 2023; Ilahi et al., 2026; Xie et al., 2021). These findings together with the results of the present study indicate that CaCl₂ treatment represents an effective strategy for preserving the color quality, lightness, and structural integrity of peaches during cold storage.

Furthermore, CaCl₂ treatment has been reported to effectively maintain the L value of fruits and vegetables during storage. In peaches and lychees, CaCl₂ reduces browning and preserves lightness by reinforcing cell wall structure and suppressing oxidative enzyme activity (Ahmad et al., 2024; Guo et al., 2023). Similar outcomes have been observed in mangoes and frozen strawberries, where CaCl₂ treatment maintained fruit brightness and natural color while minimizing total color change (ΔE) (Alharaty & Ramaswamy, 2020; Saikaew et al., 2023). In citrus fruits, CaCl₂ application delayed peel discoloration and preserved marketable appearance (Frempong et al., 2022). Studies on tomatoes and radishes have also demonstrated that spraying with 2% CaCl₂, or its combination with a starch–glycerol coating, maintained high L^⁎^ values throughout 20–56 days of storage (Mazumder et al., 2021; Oz & Akyol, 2020; Shehata et al., 2021). Overall, previous studies suggest that the protective role of CaCl₂ may be associated with the strengthening of cell wall structure and the suppression of oxidative enzyme activity.

### 3.4 TA index

TA is recognized as one of the primary post-harvest quality indicators of fresh fruits, playing a decisive role in flavor perception and consumer acceptance by regulating the balance between organic acids and sugars, thereby influencing product shelf life (Huang et al., 2021). According to the response surface model, variations in the TA of peach fruit were found to be strongly dependent on the interaction between storage duration and CaCl₂ concentration (Fig. 5). TA values ranged from 0.65% to 1.25%. The highest TA value (1.25%) was observed before storage, whereas the lowest value (0.65%) was recorded in the untreated control after 40 days of storage. Under the same 40-day storage period, fruits treated with 3% CaCl₂ retained a TA value of 0.78%, which was approximately 20% higher than that of the untreated control. This pattern may be related to respiratory metabolism and the gradual utilization of organic acids during post-harvest ripening (Zheng et al., 2021); however, these underlying processes were not directly evaluated in the present study.

**Fig. 5:**
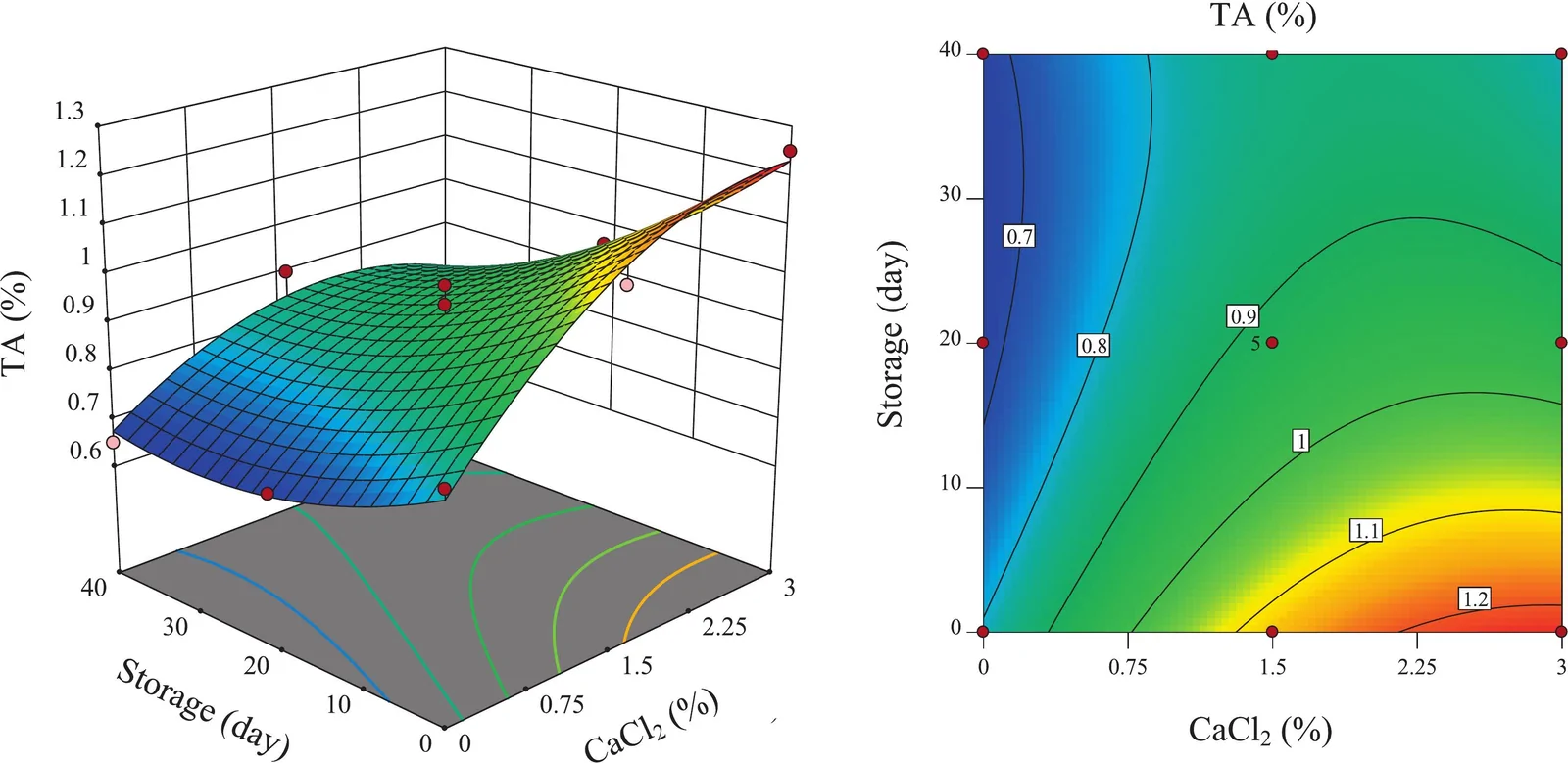
Variation in titratable acidity (TA) of peach fruit in response to CaCl₂ application and storage period.

Temporal trend analysis revealed that TA decreased over time under all treatment levels; however, the rate of decline varied depending on the CaCl₂ concentration (Fig. 5). At low doses, a mild reduction was detected, suggesting limited effectiveness of the CaCl₂ treatment in suppressing catabolic activity. In contrast, at optimal concentrations, although the initial TA level was higher, a more pronounced decrease from the starting value occurred during extended storage. This phenomenon may be associated with the gradual attenuation of the protective effects of CaCl₂’s protective effects over time and the accelerated conversion of organic acids in fruits with higher initial acid reserves (Tagele et al., 2022). Therefore, storage duration remains the dominant factor influencing TA reduction and requires precise management.

From the perspective of CaCl₂ concentration, increasing this factor during short-term storage significantly enhanced TA. It maintained higher acid reserves, possibly due to mechanisms reported in previous studies, including reduced skin permeability, stabilization of cell wall structures, and restriction of respiratory pathways (Erogul et al., 2024; Nguyen et al., 2020; Shehata et al., 2021); however, these underlying mechanisms were not directly evaluated in the present study. Although the improvement in acidity retention was less pronounced during prolonged storage, TA levels remained consistently higher than those of the control samples (Fig. 5). These findings align with previous studies on strawberries and tomatoes, which have reported a role for CaCl₂ in slowing acidity loss during cold storage (Erogul et al., 2024; Nguyen et al., 2020; Shehata et al., 2021). Overall, it can be concluded that the greatest efficacy of this compound is observed during the early stages of storage. At the same time, its sustained presence continues to act as an effective protective factor in maintaining the post-harvest quality of peaches.

### 3.5 A^⁎^/b^⁎^ index

The a^⁎^/b^⁎^ index is recognized as a key indicator for assessing color transformation and ripening stages in peaches, representing the ratio of red to yellow hues on the fruit skin, which is directly linked to consumer acceptance and market value (Kapoor et al., 2022). According to the response surface analysis, this index was significantly influenced by the interaction between CaCl₂ concentration and storage duration (Fig. 6). The a^⁎^/b^⁎^ values ranged from 0.27 to 0.65, indicating a transition from green to red coloration due to the enhancement of biochemical processes associated with ripening (Chattopadhyay et al., 2021). The lowest value (0.27) was observed in the untreated control before storage, whereas the highest value (0.65) was recorded in the untreated control after 40 days of storage. Under the same 40-day storage period, fruits treated with 3% CaCl₂ had an a^⁎^/b^⁎^ value of 0.45, which was approximately 30.8% lower than that of the untreated control. The highest a^⁎^/b^⁎^ values were recorded under prolonged storage without calcium treatment, suggesting accelerated pigmentation in the absence of protective agents. In contrast, shorter storage periods and higher CaCl₂ concentrations were associated with a significant decline in this index.

**Fig. 6:**
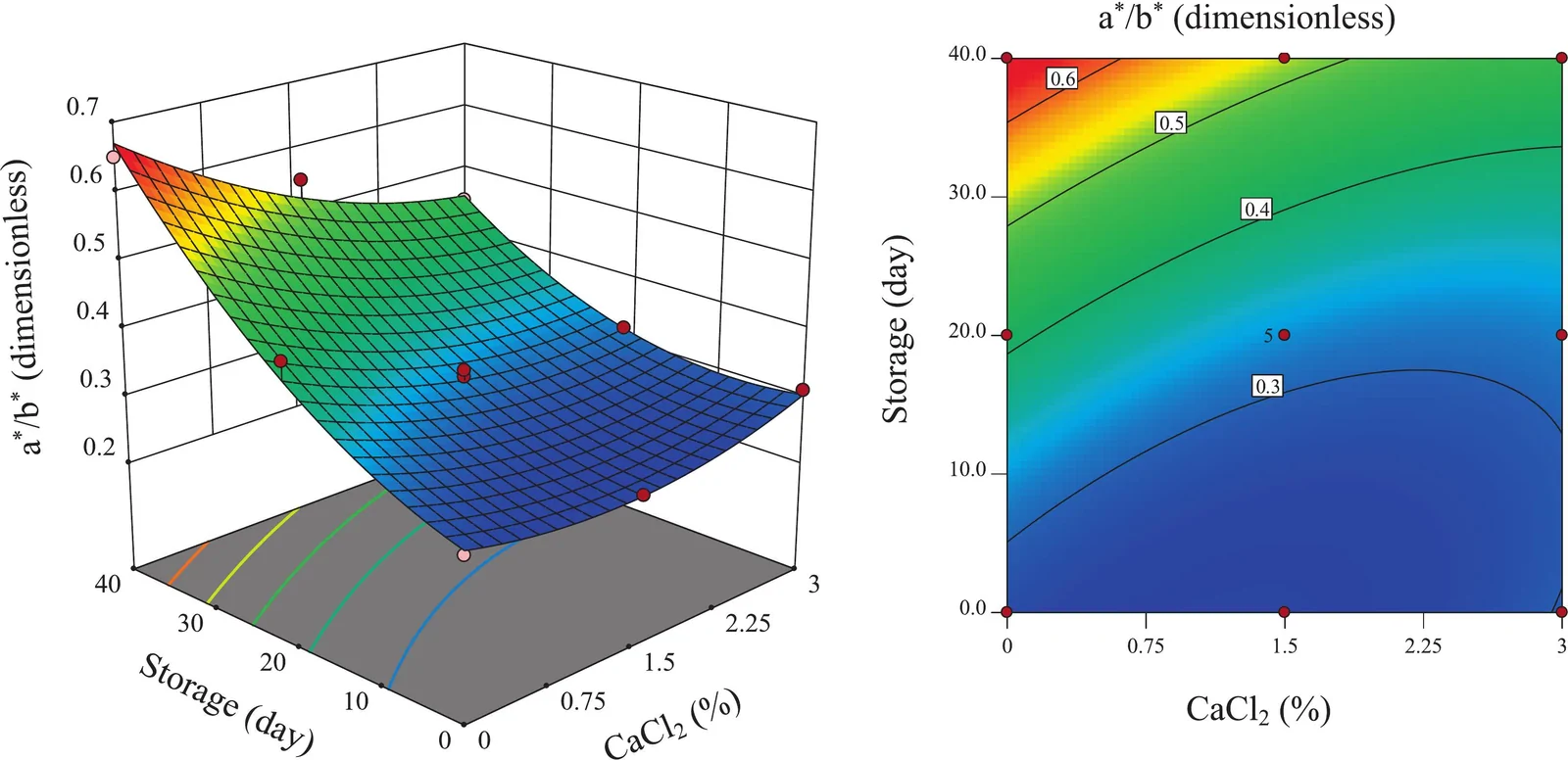
Response surface illustrating the effect of CaCl₂ treatment and storage duration on the color ratio (a^⁎^/b^⁎^) of peach fruit.

The effect of storage time indicated that the a^⁎^/b^⁎^ index increased with prolonged storage across all CaCl₂ concentrations; however, the rate of increase was strongly dependent on the pre-harvest foliar CaCl₂ treatment (Fig. 6). At low CaCl₂ concentrations, the rapid rise in a^⁎^/b^⁎^ was suggested in previous studies to be associated with progressive chlorophyll degradation and anthocyanin accumulation as a result of activated oxidative enzymatic pathways (Mattus-Araya et al., 2022). Conversely, under higher CaCl₂ concentrations, this increase occurred at a much slower rate, which may be associated with the reported role of calcium in alleviating physiological stress and modulating pigment synthesis pathways (Zou et al., 2023); however, these mechanisms were not directly evaluated in the present study.

A dual pattern was also observed regarding the CaCl₂ concentration (Fig. 6). During short-term storage, treatment concentration had negligible effects on the a^⁎^/b^⁎^ index, as pigmentation processes were still at early stages (Feng et al., 2022). However, at longer storage durations, increasing the CaCl₂ concentration resulted in lower a^⁎^/b^⁎^ values, suggesting an association between higher CaCl₂ concentrations and delayed red color development and ripening-related changes under the conditions evaluated (Lv et al., 2020). This behavior confirms that the use of higher calcium levels during extended storage can effectively prevent undesirable color changes and enhance the visual stability of the product.

These findings are consistent with previous studies reporting that CaCl₂ treatment maintained the b^⁎^ value and controlled the increase of a^⁎^ value in tomatoes (Shehata et al., 2021). Similarly, the application of 1% CaCl₂ reduced b^⁎^ value variations in frozen mango fruits (Saikaew et al., 2023), and alginate–CaCl₂ coatings in strawberries preserved color balance during storage (Alharaty & Ramaswamy, 2020). Therefore, based on the present results and corroborating evidence, CaCl₂ can be introduced as an effective strategy to control color changes associated with ripening and to improve the market quality of peaches during cold storage.

### 3.6 Et

Ethylene is recognized as a central hormonal regulator of fruit ripening, controlling the rate of tissue softening, coloration, and ripening-associated metabolic activities (Kou et al., 2021). Response surface analysis of ET in peach fruits indicated that ET levels were significantly influenced by the interaction between the CaCl₂ concentration (C) and cold storage duration (S) (Fig. 7). ET values ranged from 9.01 to 37.03 μmol kg^−1^ h^−1^. The lowest value (9.01 μmol kg^−1^ h^−1^) was observed in the untreated control before storage, whereas the highest value (37.03 μmol kg^−1^ h^−1^) was recorded in the untreated control after 40 days of storage. Under the same 40-day storage period, ET in fruits treated with 3% CaCl₂ reached 22.03 μmol kg^−1^ h^−1^, which was approximately 40.5% lower than that of the untreated control. The response surface exhibited a concave, non-monotonic parabolic pattern, highlighting the association between the CaCl₂ treatment and lower ET levels during storage. Taken together with the increase in a^⁎^/b^⁎^ and decline in TA during storage, the attenuation of ET by CaCl₂ provides converging evidence of delayed ripening-related changes in peach fruit under the conditions evaluated.

**Fig. 7:**
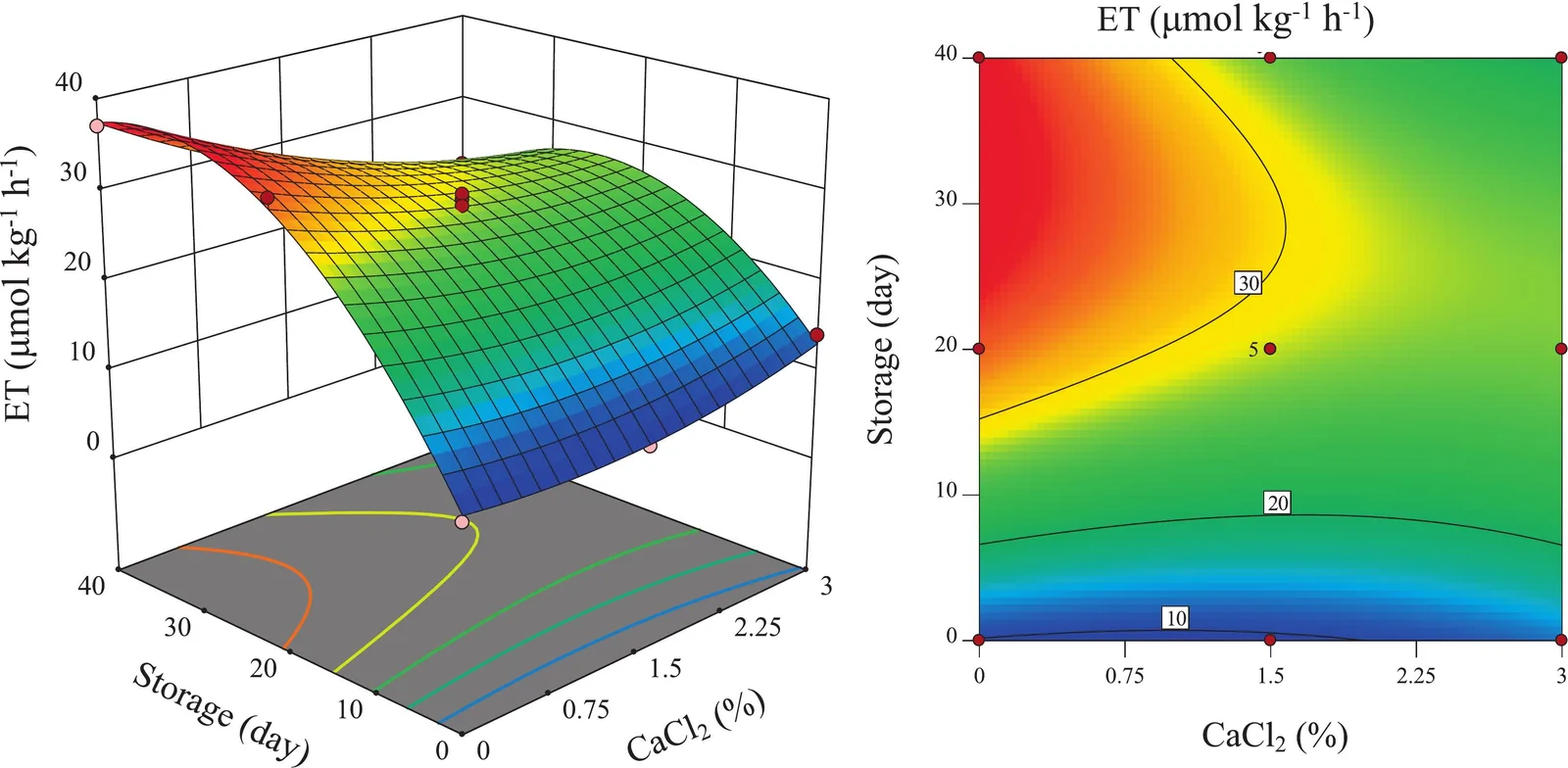
Response surface illustrating the combined influence of CaCl₂ application and storage duration on the ethylene production (ET) of peach fruit.

The effect of cold storage duration at two different CaCl₂ concentrations revealed that, at low concentrations (0–0.75%), prolonged storage led to a sharp increase in ET, which may be associated with climacteric behavior and increased activity of enzymes involved in ethylene biosynthesis, as reported previously (Li et al., 2020); however, these underlying enzymatic changes were not directly evaluated in the present study. In contrast, at higher CaCl₂ concentrations (around 3%), ET still increased with longer storage, but the rate of change was much milder and overall ET values remained lower. Previous literature suggests that the lower ET observed following CaCl₂ treatment may be associated with enhanced cell wall firmness and the modulation of ethylene biosynthesis; however, these mechanisms were not directly evaluated in the present study (Xu et al., 2023).

The influence of the CaCl₂ concentration across short and long storage periods showed that, during short-term storage (0–10 days), concentration changes had no significant effect on ET, and hormone production remained nearly constant, which is consistent with the slow initial ripening stage (Nguyen et al., 2020). However, during extended storage periods (over 30 days), higher CaCl₂ concentrations resulted in a substantial decrease in ET, suggesting that CaCl₂ may contribute to the modulation of ethylene metabolism. These findings are in agreement with earlier reports, in which CaCl₂ treatments in New Queen melons, strawberries, and radishes effectively inhibited ethylene release, the respiration rate, and tissue softening, thereby extending fruit shelf life (Alharaty & Ramaswamy, 2020; Oz & Akyol, 2020; Zhang et al., 2019). Therefore, CaCl₂ can be considered an effective strategy for reducing ethylene production and delaying ripening under the conditions evaluated in this study, consistent with previous reports.

### 3.7 AsA

AsA (Vitamin C) is recognized as an essential antioxidant and a key nutritional compound in fruits, playing a critical role in strengthening the immune system, reducing oxidative stress, and maintaining cellular health. High levels of this vitamin not only enhance the nutritional value of fruits but also improve their antioxidant capacity and protective potential against harmful agents and chronic diseases (Arabia et al., 2024). Response surface analysis of the AsA index in peach fruit indicated that vitamin C content was significantly affected by the interaction between storage duration and the CaCl₂ concentration (Fig. 8). The response surface exhibited a concave, non-monotonic parabolic pattern, indicating a nonlinear relationship between storage duration, CaCl₂ concentration, and AsA retention. AsA values ranged from 2.4 to 4.2 μg g^−1^. The lowest value (2.4 μg g^−1^) was observed in the untreated control after 40 days of storage, whereas the highest value (4.2 μg g^−1^) was recorded in fruits treated with 3% CaCl₂ after 20 days of storage. The untreated control had an AsA value of 3.7 μg g^−1^ before storage, while fruits treated with 3% CaCl₂ retained 3.4 μg g^−1^ after 40 days of storage, which was approximately 41.7% higher than that of the untreated control under the same storage conditions. Consistent with the correlation analysis presented in Fig. 2, AsA showed a strong negative correlation with a^⁎^/b^⁎^ (*r* = −0.85, *p* < 0.001), indicating a close association between AsA status and fruit color development during storage.

**Fig. 8:**
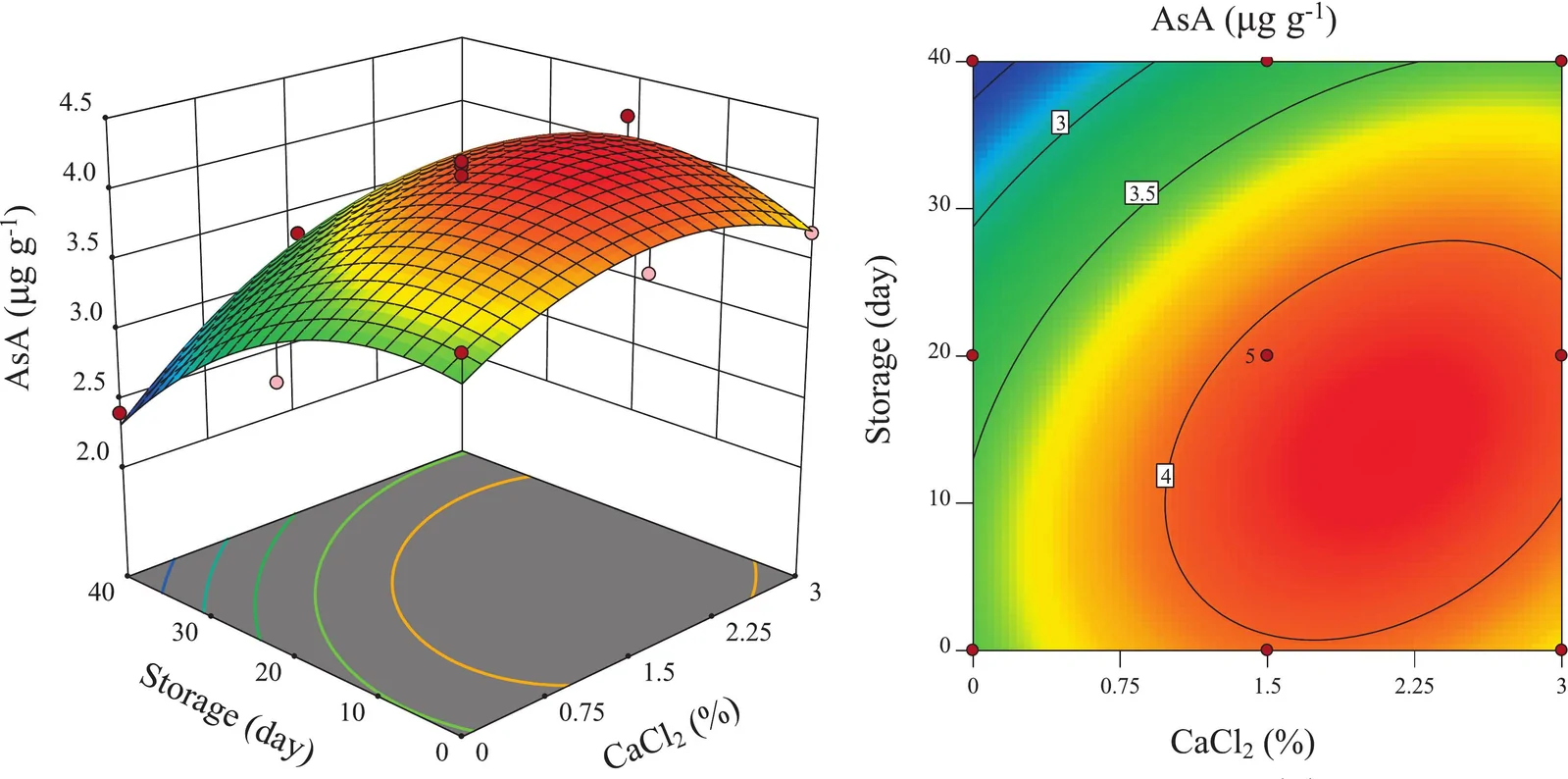
Effect of combined CaCl₂ treatment and storage period on the ascorbic acid (AsA) response surface in peach fruit.

The effect of the CaCl₂ concentration at two different storage intervals showed that during the early stages of storage, concentration changes had little influence on AsA levels (Fig. 8). This may be attributed to the natural resistance of the fruit and the stability of its biochemical system during the initial storage phase (Lv et al., 2020). However, during long-term storage, increasing the CaCl₂ concentration was found to reduce degradation and enhance relative AsA levels. According to previous reports, the observed effect may be associated with the strengthening of the cell wall structure, inhibition of oxidative enzyme activity, and reduction of the respiration rate (Feng et al., 2022); however, these mechanisms were not directly evaluated in the present study. These proposed mechanisms are consistent with previous findings and support the reported effectiveness of CaCl₂ treatment in preserving the nutritional quality and antioxidant value of fruits during extended storage periods (Guo et al., 2023; Hou et al., 2021; Liao et al., 2023).

The significant decrease in AsA under prolonged storage and at low CaCl₂ concentrations (Fig. 8) has been associated in previous studies with the activity of oxidizing enzymes and the elevated respiration rate in the absence of a protective agent (Vichaiya et al., 2020); however, these processes were not directly evaluated in the present study. These results align with similar observations in tomatoes, strawberries, and other horticultural products, supporting previous reports regarding the protective role of CaCl₂ in slowing down vitamin C degradation (Oz & Akyol, 2020; El-Mogy et al., 2020; Wahid et al., 2025). Overall, the present findings indicate that the combination of storage time and an appropriate CaCl₂ concentration is a key determinant of vitamin C stability in peach fruit. These outcomes are in agreement with reports on various agricultural products and suggest that the application of CaCl₂, particularly in combination with protective pre-harvest treatments, is an effective approach for maintaining AsA and enhancing post-harvest longevity (El-Beltagi et al., 2022; Mazumder et al., 2021).

### 3.8 Evaluation of optimal conditions and experimental validation of the model

In this study, to preserve the visual and nutritional characteristics of peach fruit during cold storage, a simultaneous optimization of two independent variables—CaCl₂ concentration and storage duration—was performed based on the desirability function. According to the first strategy, which aimed to maximize quality retention during long-term storage, an overall desirability value of 0.732 (73.2%), representing the degree to which all optimization objectives were simultaneously satisfied, was obtained at a CaCl₂ concentration of 1.96% and a storage period of 34.8 days (Fig. 9). This desirability value represents the composite degree of fulfillment of the predefined optimization criteria for the measured responses and should not be interpreted as a percentage of commercial acceptability or shelf life. Because multiple responses with different optimization directions and constraints were optimized simultaneously, the selected condition represents the best compromise identified by the RSM model within the studied design space. At this optimal point, the predicted values for L^⁎^, TA, a^⁎^/b^⁎^, ET, and AsA were 65.03, 0.87%, 0.44, 27.31 μmol kg^−1^ h^−1^, and 3.70 μg g^−1^, respectively. To further confirm the accuracy of the model predictions, an independent validation experiment was conducted in a subsequent season using newly harvested fruit from the same orchard under the predicted optimal conditions (1.96% CaCl₂ and 34.8 days of cold storage). For each optimization strategy, three uniform trees were independently selected for validation at the corresponding predicted CaCl₂ concentration and storage duration. Fruits were harvested at the same physiological maturity stage used in the main experiment, based on the defined background-color criterion, flesh firmness (70–80 N), and TSS (10–11°Brix). For each validation tree, five fruits were randomly sampled and treated as subsamples, consistent with the sampling scheme of the main experiment. The same experimental and analytical procedures were followed for the validation measurements. The resulting experimental values (Table 4) showed only minor numerical deviations from the predicted values (with a maximum deviation of 2.78%), supporting the predictive agreement of the developed regression models within the studied design space. Seasonal environmental variation could not be completely excluded. This validation confirms the predictive performance of the model for the measured responses rather than commercial shelf-life extension.

**Fig. 9:**
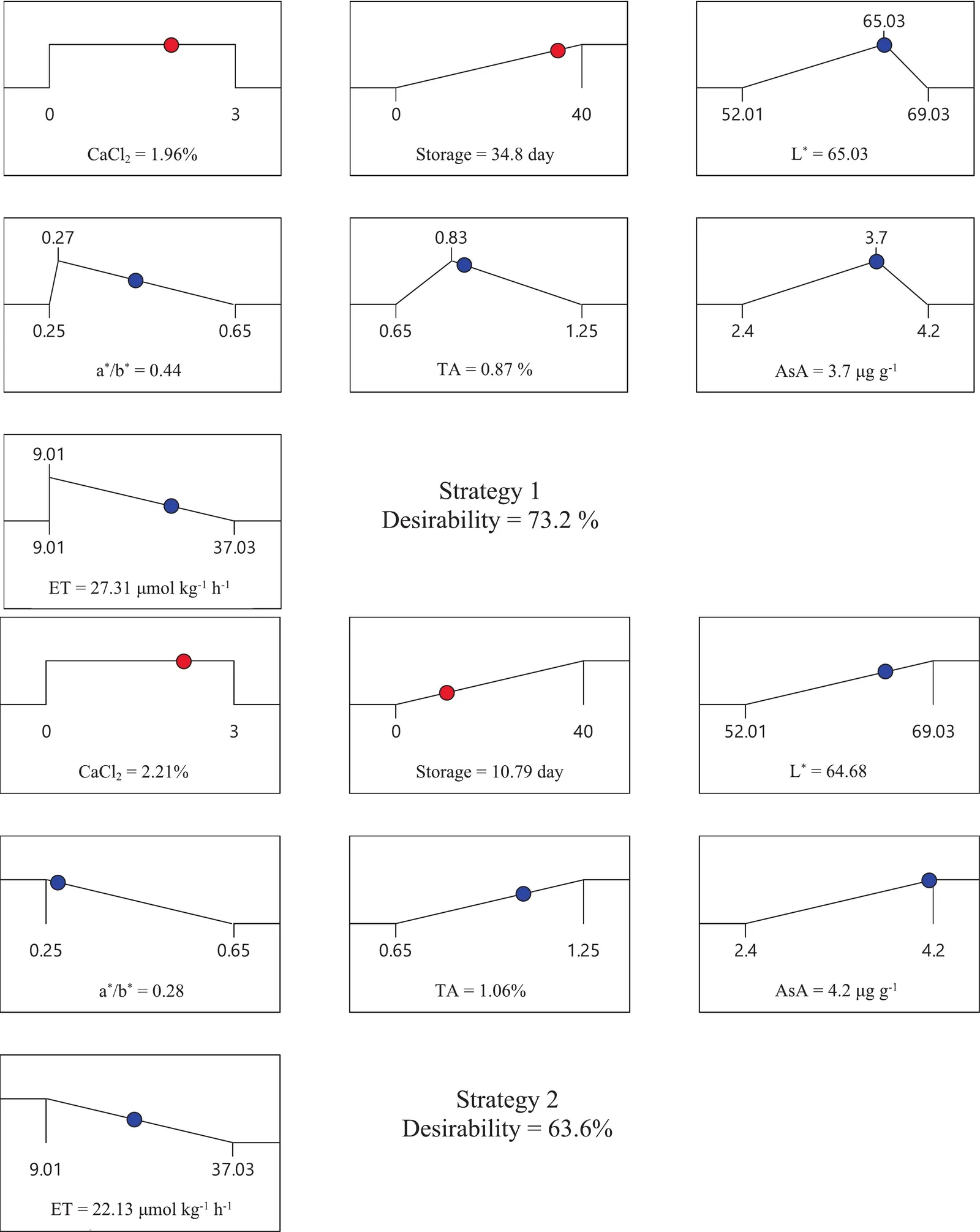
Optimized points for different CaCl₂ treatments and storage strategies for peach fruit.

**Table 4:** Experimental validation of optimized conditions.

Parameters	Optimization criteria	Predicted results	Validation
Strategy 1		Desirability: 73.2%	
CaCl_2_ (%)	In range	1.96	1.96
Storage (day)	Maximum	34.8	34.8
L^⁎^ (−)	Control value	65.03	63.22 ± 1.56
TA (%)	Control value	0.87	0.85 ± 0.009
a^⁎^/b^⁎^ (−)	Control value	0.44	0.43 ± 0.004
ET (μmol kg^−1^ h^−1^)	Control value	27.31	26.81 ± 0.04
AsA (μg g^−1^)	Control value	3.70	3.66 ± 0.11
Strategy 2		Desirability: 63.6%	
CaCl_2_ (%)	In range	2.21	2.21
Storage (day)	Maximum	10.79	10.79
L^⁎^ (−)	Maximum	64.68	62.79 ± 1.82
TA (%)	Maximum	1.06	1.03 ± 0.001
a^⁎^/b^⁎^ (−)	Minimum	0.28	0.27 ± 0.001
ET (μmol kg^−1^ h^−1^)	Minimum	22.13	21.85 ± 0.68
AsA (μg g^−1^)	Maximum	4.20	4.11 ± 0.15

In the second strategy, the objective was to simultaneously maximize or minimize each quality and nutritional parameter, resulting in a desirability value of 0.636 (63.6%). Under these conditions, the model estimated that a treatment with 2.21% CaCl₂ combined with an optimal storage duration of 10.79 days would yield L^⁎^ = 64.68, TA = 1.06%, a^⁎^/b^⁎^ = 0.28, ET = 22.16 μmol kg^−1^ h^−1^, and AsA = 4.20 μg g^−1^ (Table 4). The results from both strategies indicated that a precise adjustment of the CaCl₂ concentration combined with proper management of storage duration can significantly mitigate the decline in fruit quality.

The application of RSM in this research demonstrated its utility for optimizing storage and pre-harvest treatment conditions. This approach has been previously employed successfully for optimizing the nutritional attributes of forage maize (Hazbawi, Jahanbakhshi, Sepehr, & Ehtesham-Nia, 2025a), extracting valuable bioactive compounds (Cano-Lamadrid et al., 2023), enhancing wheat yield through integrated RSM–deep learning approaches (Godara et al., 2024), and optimizing microbial inoculants and growth regulators in agricultural plants (Hazbawi, Jahanbakhshi, Sepehr, & Ehtesham-Nia, 2025b; Emran et al., 2024). These findings support the applicability of RSM for identifying favorable combinations of treatment and storage conditions for the measured quality attributes of peach fruit.

## 4 Conclusion

The optimal adjustment of CaCl₂ concentration and storage duration effectively delayed quality deterioration in peaches during cold storage. Response surface methodology (RSM) showed that both factors significantly affected key quality attributes, including color lightness (L^⁎^), color ratio (a^⁎^/b^⁎^), titratable acidity, ethylene production, and ascorbic acid content. Pareto analysis indicated that CaCl₂ treatment contributed the most to maintaining peel brightness and overall fruit appearance. The interactive effect between treatment and cold storage conditions reduced acidity loss and preserved nutritional compounds. The optimal treatment (1.96% CaCl₂ for 34.8 days) achieved a desirability of 73.2%, and the prediction error (<2.78%) confirmed the model's reliability. Under the defined optimization criteria, this condition represented the best model-derived compromise for the measured quality attributes within the studied design space. From a practical perspective, pre-harvest CaCl₂ application shows potential for preserving selected fruit quality attributes during cold storage. Although the present study demonstrated significant improvements in physicochemical quality attributes, its findings should be interpreted within the scope of the evaluated parameters, as the underlying physiological and molecular mechanisms were not directly investigated. Furthermore, fruit firmness, decay incidence, and respiration rate were not evaluated during storage, with firmness only being assessed at harvest. Other factors relevant to broader practical or commercial assessment, including calcium uptake, yield, residue safety, sensory quality, and economic feasibility, were also not evaluated. As the study was limited to the ‘Zafarani’ cultivar and an untreated control, future research should include additional cultivars and alternative postharvest treatments, together with evaluations of fruit firmness during storage, decay incidence, and respiration rate, as well as the other practical and commercial factors noted above, to provide a more comprehensive assessment of postharvest quality.

## CRediT authorship contribution statement

**Isa Hazbawi:** Writing – review & editing, Writing – original draft, Visualization, Validation, Supervision, Software, Resources, Project administration, Methodology, Investigation, Formal analysis, Data curation.

## Declaration of competing interest

The authors declare that they have no known competing financial interests or personal relationships that could have appeared to influence the work reported in this paper.

## Data Availability

Data will be made available on request.
