# Supplementary material for: Pre-harvest CaCl₂ foliar application enhances peach quality during cold storage: Insights from response surface modeling
Source: Food Chem X. 2026 Sep 26;39:104425. doi: 10.1016/j.fochx.2026.104425 (PMC13636343; doi:10.1016/j.fochx.2026.104425)
Supplement: Supplementary file 1 [file mmc1.docx]

**Table S1**. ANOVA for the effects of CaCl₂ treatment and storage conditions on the physiological and nutritional attributes of peach fruits.

| Source | DF |  | Mean square | | | | |
| --- | --- | --- | --- | --- | --- | --- | --- |
|  |  | L^*^ | | TA | a^*^/b^*^ | ET | AsA |
| Model | 5 | 45.44^**^ | | 186.9^**^ | 0.033^**^ | 187.83^**^ | 0.62^**^ |
| CaCl_2_ treatment (C) | 1 | 112.84^**^ | | 88.5^**^ | 0.013^**^ | 66.53^**^ | 0.76^**^ |
| Storage period (S) | 1 | 16.73^*^ | | 753.5^**^ | 0.112^**^ | 468.70^**^ | 0.71^**^ |
| C × S | 1 | 72.42^**^ | | 56.6^**^ | 0.0144^**^ | 100.30^**^ | 0.25^*^ |
| C^2^ | 1 | 16.85^*^ | | 25.9^*^ | 0.005^*^ | 13.15^*^ | 0.35^*^ |
| S^2^ | 1 | 17.96^*^ | | 24.0^*^ | 0.010^**^ | 293.77^**^ | 0.51^**^ |
| Lack of fit | 3 | 1.47^ns^ | | 6.9 ^ns^ | 0.001^ns^ | 3.50^ns^ | 0.06^ns^ |
| Pure error | 4 | 3.33 | | 1.3 | 0.001 | 0.62 | 0.01 |

**Note:** DF: Degrees of freedom; ns, *, and **: Non-significant, significant at the 5% and 1% probability levels, respectively.
